# Risk factors for surgical site infection in patients undergoing colorectal surgery: A meta-analysis of observational studies

**DOI:** 10.1371/journal.pone.0259107

**Published:** 2021-10-28

**Authors:** ZhaoHui Xu, Hui Qu, ZeZhong Gong, George Kanani, Fan Zhang, YanYing Ren, Shuai Shao, XiaoLiang Chen, Xin Chen

**Affiliations:** 1 Department of Hernia and Colorectal Surgery, The Second Hospital of Dalian Medical University, Dalian, People’s Republic of China; 2 Dalian Medical University, Dalian, China; Shuguang Hospital, CHINA

## Abstract

**Objective:**

Surgical site infection (SSI) is the second most prevalent hospital-based infection and affects the surgical therapeutic outcomes. However, the factors of SSI are not uniform. The main purpose of this study was to understand the risk factors for the different types of SSI in patients undergoing colorectal surgery (CRS).

**Methods:**

PubMed, EMBASE, and Cochrane Library databases were searched using the relevant search terms. The data extraction was independently performed by two investigators using a standardized format, following the pre-agreed criteria. Meta-analysis for the risk factors of SSI in CRS patients was carried out using Review Manager 5.3 (RevMan 5.3) and Stata 15.1 software. The quality of evidence was evaluated using total sample size, Egger’s *P*-value, and intergroup heterogeneity, which contained three levels: high-quality (Class I), moderate-quality (Class II/III), and low-quality (Class IV). The publication bias of the included studies was assessed using funnel plots, Begg’s test, and Egger’s test.

**Results:**

Of the 2660 potentially eligible studies, a total of 31 studies (22 retrospective and 9 prospective cohort studies) were included in the final analysis. Eventually, the high-quality evidence confirmed that SSI was correlated with obesity (RR = 1.60, 95% confidence interval (CI): 1.47–1.74), ASA score ≥3 (RR = 1.34, 95% CI: 1.19–1.51), and emergent surgery (RR = 1.36, 95% CI: 1.19–1.55). The moderate-quality evidence showed the correlation of SSI with male sex (RR = 1.30, 95% CI: 1.14–1.49), diabetes mellitus (RR = 1.65, 95% CI: 1.24–2.20), inflammatory bowel disease (RR = 2.12, 95% CI: 1.24–3.61), wound classification >2 (RR = 2.65, 95% CI: 1.52–4.61), surgery duration ≥180 min (RR = 1.88, 95% CI: 1.49–2.36), cigarette smoking (RR = 1.38, 95% CI: 1.14–1.67), open surgery (RR = 1.81, 95% CI: 1.57–2.10), stoma formation (RR = 1.89, 95% CI: 1.28–2.78), and blood transfusion (RR = 2.03, 95% CI:1.34–3.06). Moderate-quality evidence suggested no association with respiratory comorbidity (RR = 2.62, 95% CI:0.84–8.13) and neoplasm (RR = 1.24, 95% CI:0.58–2.26). Meanwhile, the moderate-quality evidence showed that the obesity (RR = 1.28, 95% CI: 1.24–1.32) and blood transfusion (RR = 2.32, 95% CI: 1.26–4.29) were independent risk factors for organ/space SSI (OS-SSI). The high-quality evidence showed that no correlation of OS-SSI with ASA score ≥3 and stoma formation. Furthermore, the moderate-quality evidence showed that no association of OS-SSI with open surgery (RR = 1.37, 95% CI: 0.62–3.04). The high-quality evidence demonstrated that I-SSI was correlated with stoma formation (RR = 2.55, 95% CI: 1.87–3.47). There were some certain publication bias in 2 parameters based on asymmetric graphs, including diabetes mellitus and wound classification >2. The situation was corrected using the trim and fill method.

**Conclusions:**

The understanding of these factors might make it possible to detect and treat the different types of SSI more effectively in the earlier phase and might even improve the patient’s clinical prognosis. Evidence should be continuously followed up and updated, eliminating the potential publication bias. In the future, additional high-level evidence is required to verify these findings.

## Introduction

Surgical site infection (SSI), which might be either at the site of incision (superficial incisional SSI (SSSI) or deep incisional SSI (DSSI)) or any organ or space infections (OS-SSI), is a serious national health problem, affecting approximately 500,000 people in the United States each year [1]. It is the second most frequent nosocomial infection, which accounts for 40% of all the healthcare-related infections in patients undergoing surgery [2]. SSI is correlated with staying at a hospital for a long time, high readmission rates, poor quality of life, and huge healthcare costs [3–5]. It is one of the important indices of medical safety evaluation.

In general, the patients undergoing surgery, particularly those who undergo surgery for colorectal diseases, are more likely to develop SSI [6, 7]. Although their etiology is multifactorial, the majority of SSIs are preventable [8]. Multiple factors can affect the development of SSI, including patient-related factors (such as obesity, diabetes mellitus, age, gender, and smoking) and treatment-related factors (such as laparoscopic procedure, prophylactic antibiotics, and stoma creation) [5, 8–10]. Unsatisfactorily, few risk factors are generally accepted and some findings on these factors in medical literature are often contradictory. Accordingly, many perioperative interventions are supported by very limited literature evidence. More importantly, many scholars have realized that the risk factors are different for the different types of SSI. The understanding of these risk factors might better prevent and treat SSI. Besides, SSI reduces the benefits of surgical treatment. Therefore, systematically assessing the common factors of the different types of SSI is a priority. In our previous report [10], some risk factors of SSI in the patients with colorectal cancer (CRC) were presented and the associated concomitant changes and explanatory reasons were also provided. However, some patients, undergoing colorectal surgery (CRS), were excluded, which included patients with benign lesions, diverticular disease, ulcerative colitis, Crohn’s disease, volvulus, bowel obstruction, or other conditions. Therefore, this meta-analysis was conducted to review the potential risk factors of SSIs, incisional SSI (I-SSI), and OS-SSI in the patients undergoing surgery, thereby further assessing the evidence grading and aiming to offer help to clinical treatments.

## Method

The study protocol was registered with the PROSPERO database (registration ID: CRD42020178270), which is an international perspective registry for systematic reviews.

### Search strategy

Peer-reviewed literature in the PubMed, Cochrane Library and EMBASE databases were thoroughly and systematically searched using a similar strategy for each database from inception to May 2020 (cut-off date 1^st^ May 2020). The exact search strategy included searching the following mesh terms in each database: (colorectal surgery, colectom*, or proctectom*); ([colon, sigmoid, rectum, large bowel, bowel, colonic, rectal, or colorectal] and [excision, resection, surgical, surgically, surgery, or procedure]); (surgical site infection*, surgical wound infection*, or postoperative wound infection); and (risk factor*). The reference list of all the included studies was screened to extend the search. The detailed search strategy for each database is provided in the S1 Table.

### Inclusion and exclusion criteria

The study inclusion criteria were defined by PICOS (population, interventions, comparator, outcomes, and study design) categories [11].

Studies that reported the following were included in this study: (a) Patients who underwent CRS; (b) Related risk factor interventions were reported; (c) The main outcome was the incidence of SSI; (d) Studies providing effect estimates (relative risks (RRs) or odds ratios (ORs)) with corresponding 95% confidence interval (CIs). (e) Case-control or cohort studies.

Review articles, conference abstracts, unpublished gray literature, study protocols, letters, animal experiments, and studies with insufficient and overlapping data (when using the same data source and overlapping search period, there was overlapping data, which was avoided by only selecting the most recent or high-quality articles) were excluded from the current study.

### Data extraction

Two researchers (ZhaoHui Xu and Hui Qu) independently extracted the details of the included studies, including the name of the first author, publication year, year of the study, study design, study size, country, risk factors, surgical types, definition and classification of SSI, the number of patients and average follow-up time, and quality score of Newcastle–Ottawa Quality Assessment Scale (NOS). The multivariate RRs/ORs with 95% CIs were preferred rather than univariate results. Any disagreement, if found, was resolved based on the assessment of a senior investigator (Xin Chen).

### Quality assessment

Quality was assessed by scoring the 3 evaluating indicators of NOS, which included the selection of study groups, inter-comparability of groups, and outcomes, with a maximum score of 9 stars [12]. The score of each included study was also evaluated. The studies having scores of ≥6 stars were considered to be of relatively higher quality; the final results are provided in the S2 Table.

This study was conducted in strict conformity with the Meta-analysis of Observational Studies in Epidemiology (MOOSE) guidelines [13] and Preferred Reporting Items for Systematic Reviews and Meta-Analyses (PRISMA) statement [14].

### Assessment of the strength of evidence

The strength of evidence was evaluated using the total sample size >1000, Egger’s *P*-value >0.1, and intergroup inconsistency (I^2^) <50%. The Class I (high-quality) evidence referred to when the three conditions were met simultaneously. Class II (moderate-quality) and Class III (moderate-quality) evidence were defined as satisfying the two and one conditions of the three conditions, respectively. Class IV (low-quality) evidence was defined as satisfying none of these conditions [15].

### Statistical analyses

All the statistical analyses were performed using Review Manager (RevMan) software (version 5.3; The Nordic Cochrane Centre, Copenhagen, Denmark) and Stata software (version 15.1). The pooled RRs and 95% Cis from the studies were analyzed using the DerSimonian–Laird random-effects model [16]. A two-tail *P* value of 0.05 or below was considered statistically significant.

The inter-group heterogeneity was examined using Cochran’s Q (χ2) test and quantified by the I^2^ statistic. The heterogeneities were categorized into three groups based on the I^2^ value (low group <50%, moderate group 50–74%, and high group ≥75%) [17]. Sensitivity analyses were performed to recognize the potential sources of heterogeneities by changing the effect model or removing one study at a time. Publication bias was evaluated using funnel plots, Begg’s test [18], and Egger’s test [19]. The funnel plot asymmetry was further corrected using the trim and fill method.

## Results

### Study characteristics

**Fig 1:**
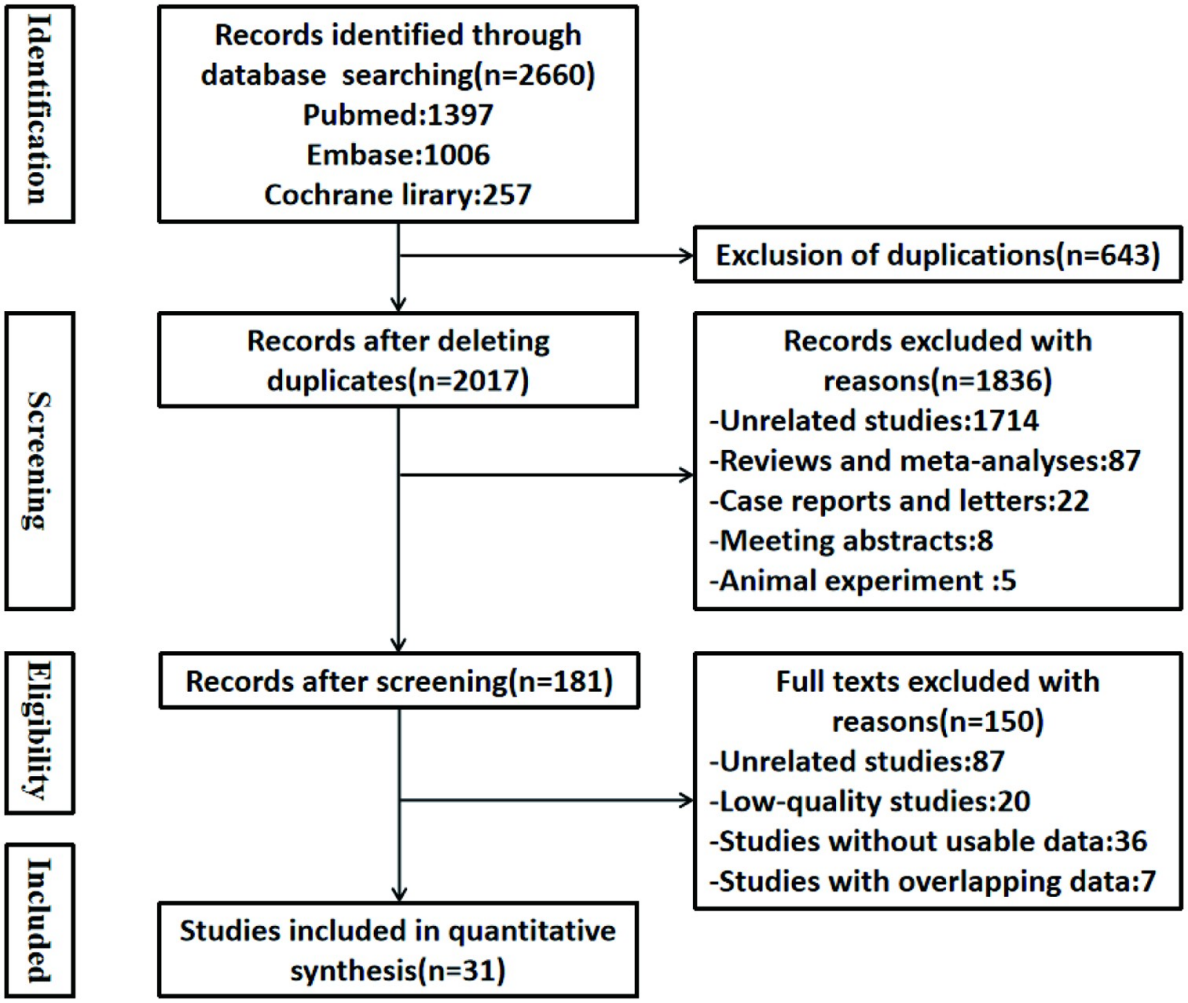
Flow chart of literature search and data extraction.

In the initial search, a total of 2660 potentially eligible studies were identified, of which 643 duplicated studies were excluded. A total of 1836 articles, including review studies, case reports, letters, animal studies, or unrelated studies, were excluded, leaving only 181 studies reviewed by the two independent investigators for full text. Thus, 31 studies were finally included. Nine of these studies were prospective cohort studies, while the remaining were retrospective cohort studies. The baseline characteristics of the included articles are presented in Table 1. The flow diagram for the study procedure is presented in Fig 1.

**Table 1:** General characteristics of the included studies in the meta-analysis.

Study	nation	Data sources	Recruited period	Procedures	No.of patients	Study type	Risk factors
Kwaan2013^38^	USA	the University of Minnesota	2008-2009	CRS	143	retrospective cohort study	1.2.29.30
Kwaan2015^35^	USA	the ACS NSQIP database	2005-2012	CRS	112,282	retrospective cohort study	1.3.4.5.6.7.8.24.25
Bot2013^21^	France	the Lille University Hospital and a private hospital	June 2004-Dec 2011	CRC	740	retrospective cohort study	4.7.8.9.10.11.16.19.20.31
Bert2017^39^	Italy	the SNICh database	Jan 2012-Dec 2012	CS	1322	retrospective cohort study	1.7.10.12
Poeran2016^25^	USA	the Premier Perspective database	Jan 2006-Dec 2013	CS	90725	retrospective cohort study	3.9.12.13.21.22.23.25.26.27
Guzman-Pruneda 2018^34^	USA	the Ohio State University Wexner Medical Center	Jan 2010-Dec 2016	CRS	469	retrospective cohort study	2.3.4.7.11.21
Ho2011^40^	USA	The NewYork-Presbyterian Hospital /Weill Cornell Medical Center	June 2001-July 2008	CRS	650	retrospective cohort study	1.6.7.10.11
Nakamura2008^41^	Japan	the Kitasato University Hospital	Jan 2004-Dec 2005	CRC	144	retrospective cohort study	7
Hennessey2015^42^	Ireland	3 institutions	2007–2009	CRS	386	retrospective cohort study	7.12.14.25
Uchino2013^43^	Japan	the Hyogo College of Medicine	Jan 2008-Dec 2011	CD	405	prospective cohort study	11.25
Tang 2001^32^	China	the Chang Gung Memorial Hospital	Feb 1995-Dec 1998	CRS	2809	prospective cohort study	11.13.15.16
Biondo2012^28^	Spain	the Spanish Rectal Cancer Project	May 2006-May 2009	RC	2131	retrospective cohort study	7.10.13.15.18.25
Bislenghi2019^20^	Belgium	the University Hospitals Leuven	Oct 2016-Jan 2017	CRS	287	prospective cohort study	5.9.14.21.37
Itatsu2013^44^	Japan	19 affiliated hospitals	Nov 2009-Feb 2011	CRC	1980	prospective cohort study	1.11.28.32.33
Hibbert2015^23^	Saudi Arab	the King Faisal Specialist Hospital & Research Centre	not involved	CRS	296	prospective cohort study	9
Hubner2011^29^	Switzerland	9 secondary and tertiary care public Swiss hospitals,	Mar 1998-Dec 2008	CS	2393	prospective cohort study	7.12.13.14
Wick2011^27^	USA	8 different BCBS insurance plans	Jan 2000-Dec 2008	CRS	7020	retrospective cohort study	7.9.13
Blumetti2007^45^	USA	a single tertiary care institution	Jan 2002-Dec 2005	CRS	428	retrospective cohort study	11.12
Tserenpuntsag2014^26^	USA	the 174 NYS hospitals	2009–2010	CS	2656	retrospective cohort study	9.10.13.15.27.38
Imai2008^30^	Japan	the Keio University Hospital	Aug 1997-Dec 2005	CC	801	retrospective cohort study	4.7.10.13.
Colas-Ruiz 2018^46^	Spain	the HUFA in Madrid	Jan 2013-Dec 2016	RS	154	prospective cohort study	15.16.21
Park 2015^36^	Korea	the Kyung Hee University Hospital, Gangdong	Jan 2010-May 2014	CRC	327	retrospective cohort study	4.5.7.10.14.15.19.35
Silvestri2017^37^	Italy	the University Hospital of Trieste	June 2010-July 2014	CRS	687	retrospective cohort study	1.4.12
Cima2017^22^	USA	the Mayo Clinic Hospital	Apr 2006-June 2014	CRS	2376	retrospective cohort study	3.4.7.9.10.17.22
Watanabe2015^47^	Japan	the Nippon Medical School Musashikosugi Hospital	July 2005-May 2010	CRS	538	retrospective cohort study	1.7.10
Mason2016^31^	UK	the Colchester University Hospital	Sep 2012-July 2014	CRS	246	retrospective cohort study	3.4.13.18.21.39
Mik2016^24^	Poland	the Medical University of Lodz and the Centre for Treatment of Bowel Diseases Hospital in Brzeziny	Jan 2008-Dec 2015	CRC	2240	retrospective cohort study	6.9.12.14.25
Olmez2019^48^	Turkey	the Kosuyolu Resarch and Education Hospital	Jan 2013-July 2019	CRC	209	retrospective cohort study	7.14
Uchino2009^49^	Japan	the Hyogo College of Medicine	Mar 2006-Dec 2007	CRS	562	prospective cohort study	10.11.22.25.36
Ghuman2015^33^	Canada	The St. Paul’s Hospital	Dec 2012-July 2014	CS	205	retrospective cohort study	3.4
Poon 2009^50^	China	the Queen Mary Hospital,	Jan2002-Dec 2006	CRC	1011	prospective cohort study	7.15.34

***Remark*:** 1. Wound classification≥3; 2.Oral antibiotics; 3.Cigarette smoking; 4.Diabetes mellitus; 5.Pulmonary comorbidities; 6.Radiation therapy; 7.Open vs minimally invasive surgery (MIS); 8.Advanced tumors; 9.Obesity; 10.ASA grade ≥3; 11.Ostomy creation; 12.Emergent surgery; 13.Male gender; 14.Operation time (≥180 min); 15. Blood transfusion; 16.Intra-abdominal drain; 17.Steroid use; 18.Converted to open procedure; 19.Hemoglobin level<10g/dL; 20.Blood loss≥500 mL; 21.Neoplasm; 22.Inflammatory Bowel Disease; 23.Diverticular Disease; 24.Cardiac comorbidity; 25.Resection procedure (Abdominoperineal resection, pelvic exenteration, extended resection, etc); 26.Hospital location; 27.Hospital Teaching Status; 28.Chronic liver disease; 29.Abdominal wall thickness (AW2); 30.History of soft tissue infection; 31.Malnutrition; 32. Previous laparotomy; 33.Wound length; 34. Anastomotic leakage; 35. Estimated blood loss (≥100 mL); 36. Preoperative hospital stay>6 days; 37. Preoperative stoma; 38.Bed size>500 vs ≤500; 39. Use of CO2 conditioner.

***Abbreviations*:** BCBS: Blue Cross and Blue Shield; CC: colon cancer; CD: Crohn’s disease; CRC: colorectal cancer; CRS: colorectal surgery; CS: colon surgery; HUFA: Hospital Universitario Fundación Alcorcón; NYS: New York State; RC: rectal cancer; RS: rectal surgery; SNICh: the National System of Surveillance of Surgical Site Infections.

### Risk factors of SSI

A total of 39 risk factors were found from the selected 31 articles. Among them, 25 factors could not be quantitatively analyzed in the study without adequate data sources and were excluded. Finally, 14 risk factors, reported in more than 2 studies, were included in this study, on which the meta-analysis was performed.

### Unmodifiable factors

#### Male sex

**Fig 2:**
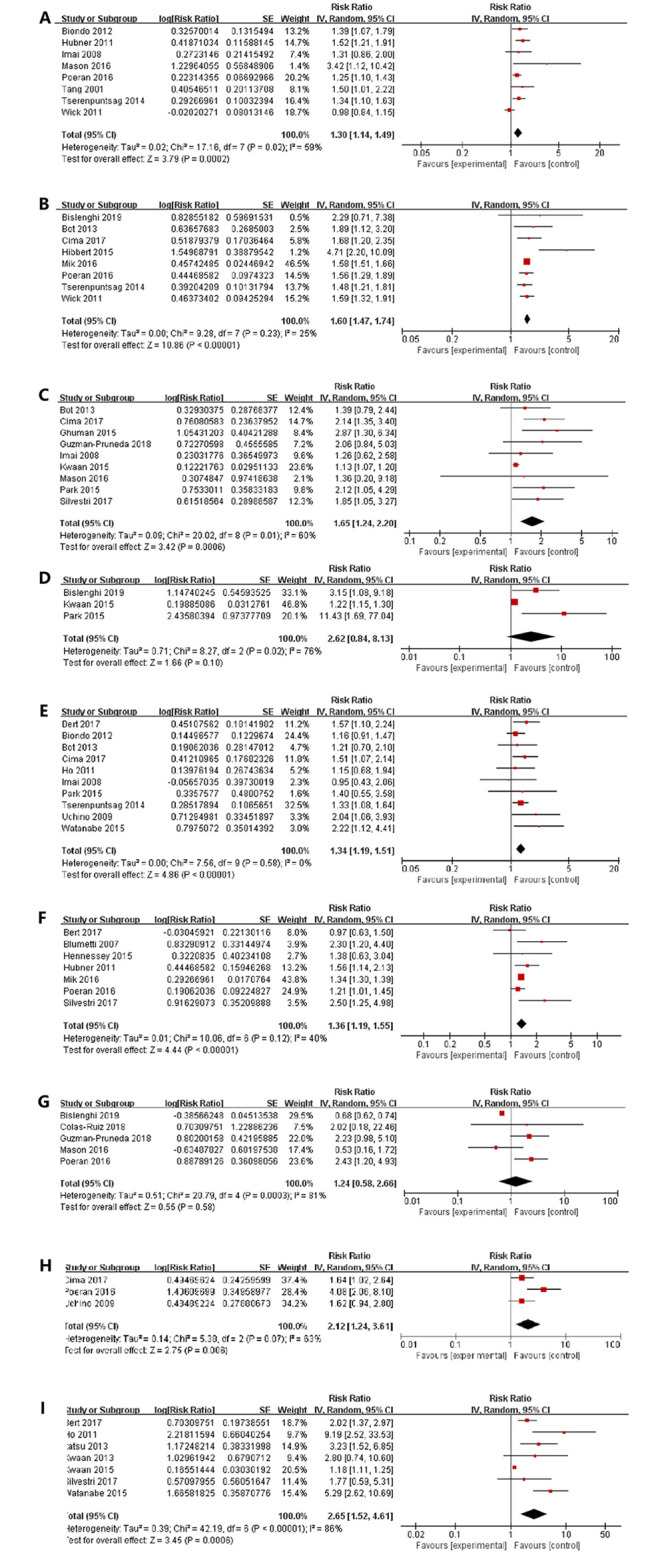
The forest plots showed that the correlations between the risk of SSIs with (A) male sex, (B) obesity, (C) diabetes mellitus, (D) respiratory disease, (E) ASA classification, (F) emergent status, (G) neoplasm, (H) inflammatory bowel disease, (I) wound classification>2.

Eight studies [5, 9, 20–25] were identified in the present study, showing that the male sex was a statistically significant risk factor for SSI (RR = 1.30, 95% CI: 1.14–1.49, I^2^ = 59%) (Fig 2A).

#### Obesity

The World Health Organization (WHO) definition for obesity was used, which defines obesity as body mass index (BMI) greater than 30 kg/m^2^ [26]. A meta-analysis of the eight studies [21, 23, 24, 27–31], which reported obesity, showed that the obese patients were positively correlated with the rate of SSI (RR = 1.60, 95% CI: 1.47–1.74, I^2^ = 25%) (Fig 2B).

#### Diabetes mellitus

Nine studies [20, 25, 27, 30, 32–36], including 118,133 patients, showed that there was a positive linear proportional correlation between the diabetes mellitus and rate of SSIs in the patients undergoing CRS (RR = 1.65, 95% CI: 1.24–2.20, I^2^ = 60%) (Fig 2C).

#### Respiratory comorbidity

Three studies [31, 33, 34] reported the connection between respiratory comorbidity and SSI. The synthetic results of these studies showed that there was a significant correlation between them in the patients undergoing CRS (RR = 2.62, 95% CI: 0.84–8.13, I^2^ = 76%) (Fig 2D).

#### American Society of Anesthesiologists (ASA) classification

Among ten studies [20, 22, 23, 27, 30, 34, 37–40], the combined results of meta-analysis revealed that the ASA score of higher than or equal to 3 showed an increased risk of developing SSI (RR = 1.34, 95% CI: 1.19–1.51, I^2^ = 0%) (Fig 2E).

#### Emergent surgery

The results of seven studies [5, 24, 29, 35, 40–42] showed that the emergent status could increase the risk of SSI by 36% (RR = 1.36, 95% CI: 1.19–1.55, I^2^ = 40%) (Fig 2F).

#### Neoplasm

The meta-analysis of five studies [24, 25, 31, 36, 43] found that there was not a significant correlation between neoplasm and SSI (RR = 1.24, 95% CI: 0.58–2.26, I^2^ = 81%) (Fig 2G).

#### Inflammatory Bowel Disease(IBD)

The meta-analysis of three studies [24, 30, 37], reporting IBD, indicated that IBD could increase the SSI rate (RR = 2.12, 95% CI: 1.24–3.61, I^2^ = 63%) (Fig 2H).

#### Wound classification>2

The association between wound classification and SSI was investigated in seven studies [33, 35, 38–40, 44, 45]. The pooled results indicated that the wound classification >2 might increase the occurrence of SSI (RR = 2.65, 95% CI: 1.52–4.61, I^2^ = 86%) (Fig 2I).

### Modifiable factors

#### Operative time (≥180 min)

**Fig 3:**
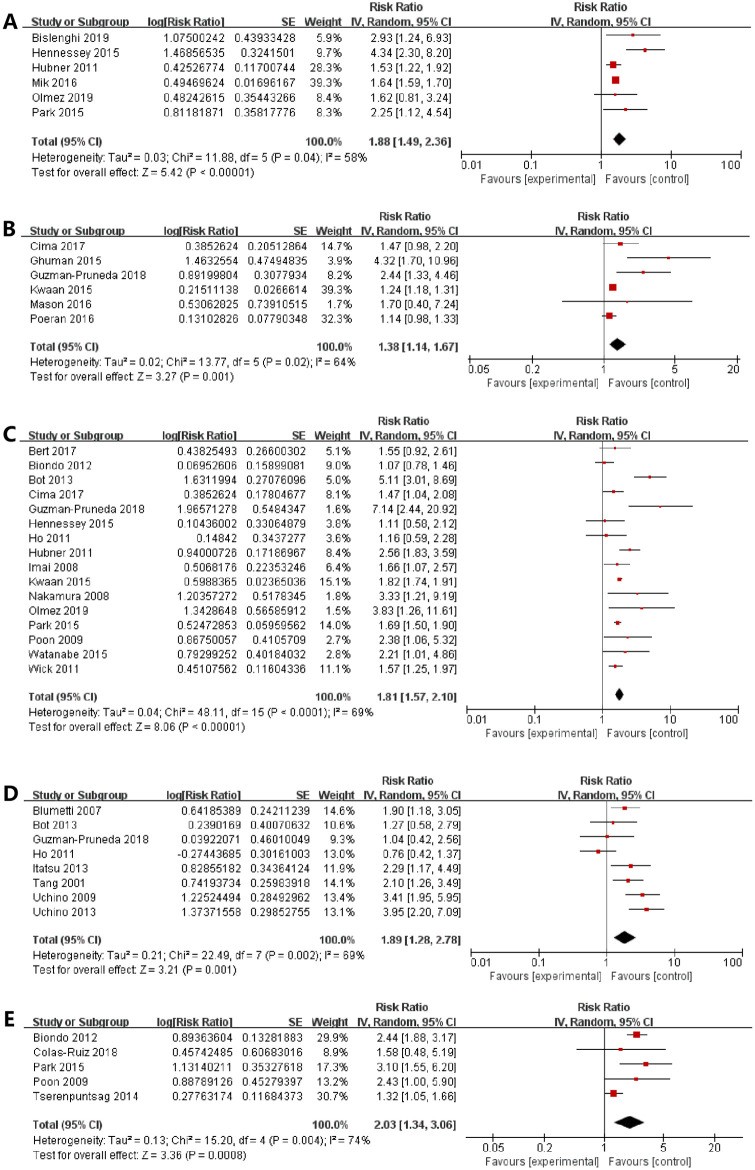
The forest plots showed that the correlations between the risk of SSIs with (A) operative time (≥180min), (B) cigarette smoking, (C) open surgery, (D) stoma formation, (E) blood transfusion.

Six studies [5, 29, 31, 34, 42, 46] focused on the effects of surgery duration. There was an 88% increase in the risk of SSI for the surgeries having the duration of longer than 180 min (RR = 1.88, 95% CI: 1.49–2.36, I^2^ = 58%) (Fig 3A).

#### Cigarette smoking

The pooled data from six studies [24, 25, 30, 32, 33, 36] showed that the smoking patients had a 1.22-fold increased risk of developing SSI as compared to the non-smoking patients (RR = 1.38, 95% CI: 1.14–1.67, I^2^ = 64%) (Fig 3B).

#### Open surgery

A meta-analysis of sixteen studies [5, 20–22, 27, 30, 33, 34, 36, 38–40, 42, 46–48] showed that the patients who accepted laparotomy had a 1.81-fold increased risk of developing SSI as compared to the patients with laparoscopic surgery (RR = 1.81, 95% CI: 1.57–2.10, I^2^ = 69%) (Fig 3C).

#### Stoma formation

The pooled analysis of eight studies [9, 27, 36–38, 41, 45, 49] suggested that the risk of SSI in the patients having in-hospital stoma formation was 1.89 times higher than those who did not have one (RR = 1.89, 95% CI: 1.28–2.78, I^2^ = 69%) (Fig 3D).

#### Blood transfusion

The pooled results of five studies [22, 23, 34, 43, 48] indicated that the perioperative blood transfusion increased the risk of developing SSIs by 103% (RR = 2.03, 95% CI:1.34–3.06, I^2^ = 74%) (Fig 3E).

### Risk factors of I-SSI and OS-SSI

The risk factors for OS-SSI were also identified (Table 2). These risk factors included: obesity (RR = 1.63, 95% CI: 1.48–1.80, *P* <0.00001); ASA score ≥3 (RR = 1.14, 95% CI: 0.90–1.46, *P* = 0.28); open surgery (RR = 1.37, 95% CI: 0.62–3.04, *P* = 0.44); stoma creation (RR = 1.19, 95% CI: 0.95–1.49, *P* = 0.12); and blood transfusion (RR = 2.32, 95% CI: 1.26–4.29, *P* = 0.007).

**Table 2:** Risk factors of SSIs, I-SSI, and O-SSI in patients undergoing CRS.

Significant factors	No. of studies	No. of patients	I2(%)	PEgger’S	P_Begg-Mazumdar’S_	RR	Evidence grading
SSIs							
Male sex	8	109727	59	0.106	0.174	1.30(1.14–1.49)	Class II (moderate-quality)
Obesity	8	106,340	25	0.161	0.063	1.60(1.47–1.74)	Class I (high-quality)
Diabetes mellitus	9	118,133	60	0.006	0.971	1.65(1.24–2.20)	Class III (moderate-quality)
ASA score≧3	10	13,049	0	0.415	0.474	1.34(1.19–1.51)	Class I (high-quality)
Emergent surgery	7	98,181	40	0.55	0.548	1.36(1.19–1.55)	Class I (high-quality)
IBD	3	93,663	63	0.262	0.296	2.12(1.24–3.61)	Class II (moderate-quality)
Wound classification	7	117,602	86	0.007	1.000	2.65(1.52–4.61)	Class III (moderate-quality)
Operative Time(≧180min)	6	5,842	58	0.195	0.133	1.88(1.49–2.36)	Class II (moderate-quality)
Cigarette smoking	6	206,303	64	0.129	0.260	1.38(1.14–1.67)	Class II (moderate-quality)
Open surgery	16	133,745	69	0.707	0.137	1.81(1.57–2.10)	Class II (moderate-quality)
Stoma creation	8	8,043	69	0.424	0.536	1.89(1.28–2.78)	Class II (moderate-quality)
Blood transfusion	5	6,279	74	0.567	0.806	2.03(1.34–3.06)	Class II (moderate-quality)
Respiratory comorbidity	3	112,896	76	0.079	0.296	2.62(0.84–8.13)	Class III (moderate-quality)
Neoplasm	5	91,881	81	0.466	0.462	1.24(0.58–2.26)	Class II (moderate-quality)
I-SSI							
Stoma	5	5,933	0	0.197	0.221	2.55(1.87–3.47)	Class I (high-quality)
O-SSI							
Obesity	3	7,272	17	0.002	0.296	1.63(1.48–1.80)	Class II (moderate-quality)
Blood transfusion	3	5,215	86	0.900	1.000	2.32(1.26–4.29)	Class II (moderate-quality)
ASA score≧3	3	5,157	0	0.821	1.000	1.14(0.90–1.46)	Class I (high-quality)
Open surgery	3	5,157	84	0.676	1.000	1.37(0.62–3.04)	Class II (moderate-quality)
Stoma creation	3	3,452	15	0.292	0.296	1.19(0.95–1.49)	Class I (high-quality)

The risk factors for developing I-SSI were also explored. It was found that the risk factors of each study were not completely consistent (S3 Table). The classifications were not completely consistent even for the same indicators but still, the stoma creation was found to be a statistically significant risk factor for developing I-SSI (Table 2) (RR = 2.55, 95% CI: 1.87–3.47, *P* <0.05).

### Sensitivity analyses

Further sensitivity analyses were conducted due to varying degrees of heterogeneities in the study. The merging direction of any risk factor was not significantly influenced using the fixed-effect models or random-effects (S4 Table). The pooled RR for the remaining studies remained unchanged in the above analysis after sequentially omitting any single study (S5 Table). Only the removal of Kwaan’s [33] study from the analysis of respiratory comorbidity changed the overall conclusion (RR ranged from 2.62 (95% CI: 0.84–8.13) to 4.66 (95% CI: 1.46–14.89). Consequently, the results of this study regarding all the other risk factors might be stable.

### Assessment of publication bias

The funnel plot was used to qualitatively assess the publication bias (S1 Fig). There was a publication bias in some analyses due to asymmetric graphs.

The *P-*value, in the analysis of diabetes mellitus, was less than 0.05 based on the results of Egger’s test, indicating a certain publication bias (Table 2). Accordingly, the funnel plot asymmetry was corrected using the trim and fill method. The five squared dots represented the effective quantities condition of included studies in the future and the corrected estimates of the intervention effects of 14 studies were 1.163 (95% CI = 0.901–1.501) (S2A Fig).

In the analysis of wound classification >2, the *P-*value was less than 0.05 based on the results of Egger’s test, indicating a certain publication bias (Table 2). Accordingly, the funnel plot asymmetry was corrected using the trim and fill method. The four squared dots represented the effective quantities condition of included studies in the future and the corrected estimated of intervention effects of 11studies was 1.310 (95% CI = 0.790–2.173) (S2B Fig). Taken together, the relevant evidence should be continuously followed up and updated, eliminating the potential publication bias.

## Discussion

The factors in this study could be divided into two categories: modifiable and unmodifiable factors. The clinicians should monitor SSI earlier to achieve early prevention, intervention, and even effective treatment by targeting the unmodifiable factors. For the modifiable factors, the indicators can be adjusted throughout the perioperative period to further reduce the occurrence of SSI.

The commonly investigated unmodifiable risk factors, at least in the short term, including gender, obesity, ASA score, and primary disease diagnosis, can affect the incidence of SSI. Male sex is prone to develop SSI (RR = 1.30) due to abdominal visceral obesity. This might lead to a more complicated procedure with a longer surgical duration and incision, thereby increasing the SSI rate [50]. Obesity is commonly perceived to be associated with adverse outcomes, such as SSI [50]. It is worth noting that an appropriate definition of reasonable obesity might take into account the differences in visceral fats and ethnicity [50]. As reported, the BMI and SSI might be linearly correlated [51, 52]. Interestingly, the BMI <20 kg/m^2^ is also an independent risk factor for SSI, reflecting the patient’s malnutrition [31].

For unclear reasons, in this study, IBD but not cancer was strongly correlated with SSI. Increasing studies [51, 53] have found that the types of SSI were correlated with underlying disease diagnosis. For instance, the patients with diverticular developed more SSSI. Strikingly, patients with IBD had more DSSI and OS-SSI. The intrinsic mechanism for the increased correlation of disease diagnosis with SSI has not been emphasized yet in the medical literature. However, the SSI surveillance of CRS should take the surgical site and disease classification into account to more effectively identify the risk factors and reduce the occurrence of SSI. Meanwhile, more attention should be paid to the patients having one or more of the above risk factors in the postoperative follow-up period.

In this study, some modifiable factors also merit attention. The laparoscopic approach was correlated with the reduced occurrence of SSI. This has been already proven in previous studies [7, 54]. Notably, in this study, laparoscopic surgery could reduce the overall SSI rate but not OS-SSI. Unsurprisingly, this study showed that the long duration of surgery (≥180 min) was an independent predictor of SSI (RR = 1.88), which was consistent with a previous studies [31]. Moreover, the likelihood of SSI can be increased by increasing the duration of surgery [55]. The long-duration [56] is usually a reliable symbol of the complexity of the surgical procedure, with possible accidental local tissue injuries. A previous study [57] showed that the in-hospital stoma formation was a risk factor for SSSI and DSSI but that study did not investigate OS-SSI. Similarly, this study found an 89% increase in the risk of SSI in patients having in-hospital stoma formation. The colostomy closure could lead to SSI in the patients having CRS [58, 59]. Perioperative blood transfusion is related to immunomodulation that could explain the increase in SSI rate [43]. Therefore, it is advised for the clinicians to focus on their surgical skills, shorten surgery duration, and reduce intraoperative blood loss and perioperative blood transfusion. In the meantime, the clinicians should suitably control the indicators of stoma formation and avoid unnecessary ones.

Cigarette smoking can delay wound healing, even for a minor and clean wound, thereby increasing the risk of SSI [8, 33, 36, 60]. This study found that the smokers had a 1.38-fold increased risk of developing SSI in comparison with the nonsmokers, which was consistent with the NNIS guidelines [58]. Smoking cessation instead of decreasing the level of smoking should be a routine as a part of perioperative management but there is often a time-limitation. Four weeks of abstinence from smoking before surgery might be appropriate [60]. A standard time to achieve smoking cessation as a part of perioperative management needs to be evaluated in more prospective studies.

The existence of underlying basic diseases can easily lead to the occurrence of SSI. Numerous studies [6, 61, 62] have confirmed that postoperative hyperglycemia is an independent risk factor for SSI and is also independent of diabetes. The uses of perioperative glycemic controls vary by country in the patients undergoing surgery. Many patients might adjust according to their actual situation. Further prospective studies are needed to verify the ideal perioperative glycemic regimens and optimal hemoglobin A1C target levels. Respiratory diseases are found to be associated with poor postoperative outcomes after CRS [63] but its correlation with SSI was not found in this study.

It is not appropriate to perform only preoperative mechanical bowel preparation in the patients undergoing CRS. Many antibiotic regimens have been studied to prevent SSI in patients undergoing CRS but there is no consensus on which antibiotic is the best [38]. Mechanical and oral antibiotic bowel preparation is widely used to reduce the risk of SSI after CRS [1, 64–66], which is well-accepted among many clinicians. Microorganisms in the intestinal lumen during surgery are still the potential infection sources of the surgical area; a joint plan is essential. On contrary, this view is suspected by more than 50% of American clinicians [67]. There are even calls for a reconsideration of this recommendation [68]. It should also be noted that the type of bowel preparation regimens cannot replace intravenous prophylactic antibiotics preoperatively. Many guidelines [69, 70] usually state that it should be administered within 60 min of an incision. The intra-operative re-dosing depends on the half-life of the drug and can be extended up to postoperative 24 h but this recommendation has not been tested rigorously. Owing to the lack of data, this aspect was not analyzed in this study.

There were different degrees of heterogeneities among the included studies, which were due to the differences in various clinical factors and parameters. First, the specific surgical procedures and the surgeon’s surgical skills in each study were not completely consistent. Second, there were differences in cultural backgrounds. There were more or fewer differences in age, sex, education level, and national region among the patients in each trial. Thirdly, the methodological heterogeneity was caused by different studies. Nevertheless, this reflected a real-life situation and the results were trustworthy.

This study will be useful in future studies regarding SSI. This study aimed to provide data to solidify some risk factors but there are still some shortcomings. First, an important factor in preventing SSI is the surgeon’s competence and skills [71], which is a variable factor that is difficult to quantify. The surgeon’s experience could not be quantified in this study. Secondly, there might be inherent bias due to the nature of retrospective or prospective cohort studies. Therefore, more studies and randomized controlled trials are still needed. Thirdly, the information of all the risk factors for I-SSI and OS-SSI could not be integrated due to the non-identical factor profiles of different studies. These differences showed that the two subtypes might have distinct pathogenesis and risk factors.

## Conclusions

The study showed that 12 factors (male sex, diabetes mellitus, obesity, ASA score ≥3, cigarette smoking, wound classification >2, IBD, open surgery, stoma formation, emergent surgery, operative time ≥180 min, and perioperative blood transfusion) were the significant risk factors for SSI. Moreover, two factors (obesity and blood transfusion) and one factor (stoma formation) were the significant risk factors for OS-SSI and I-SSI, respectively. A better understanding of these issues can lead to carrying out the precise intervention. There were some certain publication bias in 2 parameters based on asymmetric graphs, including diabetes mellitus and wound classification >2. Evidence should be continuously followed up and updated, eliminating the potential publication bias. In the future, additional high-level studies (such as well-designed randomized controlled trials or high-strength evidence according to the different grading systems) are needed to verify these results.

## Supporting information

S1 Table Detailed search strategies for each database. (DOC)

S2 Table Quality assessment of included studies using NOS. (DOC)

S3 Table Risk factor of I-SSI in patients undergoing CRS. (DOC)

S4 Table Sensitivity analysis of the meta-analysis. (DOC)

S5 Table Sensitivity analysis of the meta-analysis. (DOC)

S1 Fig The publication bias of the relevant factors of SSIs (A: Male sex; B: Obesity; C: Diabetes mellitus; D: ASA score≥3; E: Emergent surgery; F: Wound classification> 2; G: Operative time ≥180 min; H: Cigarette smoking; I: Open surgery; J: Stoma formation). (DOC)

S2 Fig The trim and fill graphs. (A: Diabetes mellitus; B: wound classification>2).
(DOC)

S1 Checklist (PDF)
