# Supplementary material for: Risk factors for surgical site infection in patients undergoing colorectal surgery: A meta-analysis of observational studies
Source: PLoS One. 2021 Oct 28;16(10):e0259107. doi: 10.1371/journal.pone.0259107 (PMC8553052; doi:10.1371/journal.pone.0259107)
Supplement: S1 Table — (DOC) [file pone.0259107.s001.doc]

| **S1a Table.** The search strategy in PubMed Database | | |
| --- | --- | --- |
| **Search** | **Query** | **Items found** |
| **#01** | "Colorectal Surgery"[Mesh] | 3371 |
| **#02** | Surgery Specialty, Colon and Rectal | 71469 |
| **#03** | Colon and Rectal Surgery Specialty | 71469 |
| **#04** | Colon Surgery Specialty | 71507 |
| **#05** | Specialty, Colon Surgery | 71507 |
| **#06** | Surgery Specialty, Colon | 71507 |
| **#07** | Proctology | 72084 |
| **#08** | Specialty, Rectal Surgery | 71503 |
| **#09** | Surgery Specialty, Rectal | 71503 |
| **#10** | Rectal Surgery Specialty | 71503 |
| **#11** | "Proctectomy"[Mesh] | 3707 |
| **#12** | Proctectomies | 4711 |
| **#13** | "Colectomy"[MESH] | 20472 |
| **#14** | Colectomies | 25969 |
| **#15** | #1 OR #2 OR #3 OR #4 OR #5 OR #6 OR #7 OR #8 OR #9 OR #10 OR # 11 OR #12 OR #13 OR #14 | 91257 |
| **#16** | "Colon, Sigmoid"[Mesh] | 7742 |
| **#17** | Sigmoid Colon | 16077 |
| **#18** | Sigmoid | 27436 |
| **#19** | "Colon"[Mesh] | 68046 |
| **#20** | "Rectum"[Mesh] | 39618 |
| **#21** | Rectums | 75431 |
| **#22** | Bowel | 504196 |
| **#23** | Large bowel | 160680 |
| **#24** | Rectal | 117771 |
| **#25** | Colonic | 272043 |
| **#26** | #16 OR #17 OR #18 OR #19 OR #20 OR #21 OR #22 OR #23 OR #24 OR #25 | 739950 |
| **#27** | Excision | 150740 |
| **#28** | Resection | 282659 |
| **#29** | Surgical | 3695477 |
| **#30** | Surgically | 3170823 |
| **#31** | Surgery | 4714589 |
| **#32** | Procedure | 8091009 |
| **#33** | #27 OR #28 OR #29 OR #30 OR #31 OR #32 | 10866539 |
| **#34** | #26 AND #33 | 392073 |
| **#35** | #15 OR #34 | 430457 |
| **#36** | "Surgical Wound Infection"[Mesh] | 36058 |
| **#37** | Infections, Surgical Wound | 53843 |
| **#38** | Surgical Wound Infections | 53843 |
| **#39** | Wound Infections, Surgical | 53843 |
| **#40** | Infection, Surgical Wound | 50764 |
| **#41** | Surgical Site Infection | 57389 |
| **#42** | Infection, Surgical Site | 57389 |
| **#43** | Infections, Surgical Site | 55181 |
| **#44** | Surgical Site Infections | 55181 |
| **#45** | Wound Infection, Postoperative | 55065 |
| **#46** | Wound Infection, Surgical | 50764 |
| **#47** | Infection, Postoperative Wound | 55065 |
| **#48** | Infections, Postoperative Wound | 54298 |
| **#49** | Postoperative Wound Infections | 54298 |
| **#50** | Wound Infections, Postoperative | 54298 |
| **#51** | Postoperative Wound Infection | 55065 |
| **#52** | #36 OR #37 OR #38 OR #39 OR #40 OR #41 OR #42 OR #43 OR #44 OR #45 OR #46 OR #47 OR #48 OR #49 OR #50 OR #51 | 67249 |
| **#53** | "Risk Factors"[Mesh] | 812366 |
| **#54** | Factor, Risk | 1435902 |
| **#55** | Factors, Risk | 1305587 |
| **#56** | Risk Factor | 1435902 |
| **#57** | Population at Risk | 1482845 |
| **#58** | Risk, Population at | 1482845 |
| **#59** | Populations at Risk | 1354313 |
| **#60** | Risk, Populations at | 1354313 |
| **#61** | #53 OR #54 OR #55 OR #56 OR #57 OR #58 OR #59 OR #60 | 1619185 |
| **#62** | #35 AND #52 AND #61 | 1397 |

|  | **S1b Table.** The search strategy in Embase Database |  |
| --- | --- | --- |
| **Search** | **Query** | **Items found** |
| **#01** | colorectal surgery'/exp | 22,249 |
| **#02** | surgery specialty, colon and rectal':ti,ab,kw OR 'colon and rectal surgery specialty':ti,ab,kw OR 'colon surgery specialty':ti,ab,kw OR 'specialty, colon surgery':ti,ab,kw OR 'surgery specialty, colon':ti,ab,kw OR'proctology':ti,ab,kw OR 'specialty, rectal surgery':ti,ab,kw OR 'surgery  specialty, rectal':ti,ab,kw OR 'rectal surgery specialty':ti,ab,kw | 1,062 |
| **#03** | rectum resection'/exp | 17,397 |
| **#04** | 'proctectomy':ti,ab,kw OR 'proctectomies':ti,ab,kw | 2,103 |
| **#05** | colon resection'/exp | 43,545 |
| **#06** | colectomy':ti,ab,kw OR 'colectomies':ti,ab,kw | 20,390 |
| **#07** | #1 OR #2 OR #3 OR #4 OR #5 OR #6 | 78,237 |
| **#08** | 'sigmoid'/exp | 16,933 |
| **#09** | colon, sigmoid':ti,ab,kw OR 'sigmoid colon':ti,ab,kw | 11,647 |
| **#10** | colon'/exp | 88,358 |
| **#11** | rectum'/exp | 41,777 |
| **#12** | rectums':ti,ab,kw OR 'bowel':ti,ab,kw OR 'rectal':ti,ab,kw OR 'colonic':ti,ab,kw OR 'colorectal':ti,ab,kw OR 'large bowel':ti,ab,kw | 598,987 |
| **#13** | #8 OR #9 OR #10 OR #11 OR #12 | 656,582 |
| **#14** | excision':ti,ab,kw OR 'resection':ti,ab,kw OR 'surgical':ti,ab,kw OR 'surgically ': ti, ab, kw OR 'surgery':ti,ab,kw OR 'procedure':ti,ab,kw | 3,348,804 |
| **#15** | #13 AND #14 | 198,082 |
| **#16** | #7 OR #15 | 235,618 |
| **#17** | surgical infection'/exp | 48,596 |
| **#18** | surgical wound infection':ti,ab,kw OR 'infections, surgical wound':ti,ab,kw OR 'surgical wound infections':ti,ab,kw OR 'wound infections, surgical':ti,ab,kw OR 'infection, surgical wound':ti,ab,kw OR 'surgical site infection':ti,ab,kw OR 'infection, surgical site':ti,ab,kw OR 'infections, surgical site':ti,ab,kw OR 'surgical site infections':ti,ab,kw OR 'wound infection, postoperative':ti,ab,kw OR 'wound infection, surgical':ti,ab,kw OR 'infection, postoperative wound':ti,ab,kw OR 'infections, postoperative wound':ti,ab,kw OR 'postoperative wound infections':ti,ab,kw OR 'wound infections, postoperative':ti,ab,kw OR 'postoperative wound infection':ti,ab,kw | 19,624 |
| **#19** | #17 OR #18 | 52,929 |
| **#20** | 'risk factor'/exp | 1,012,381 |
| **#21** | 'risk factors':ti,ab,kw OR 'factor, risk':ti,ab,kw OR 'factors, risk':ti,ab,kw OR 'population at risk':ti,ab,kw OR 'risk, population at':ti,ab,kw OR 'populations at risk':ti,ab,kw OR 'risk, populations at':ti,ab,kw | 661,066 |
| **#22** | #20 OR #21 | 1,239,369 |
| **#23** | #16 AND #19 AND #22 | 1,006 |

|  | **S1c Table**. The search strategy in Cochrane Databse |
| --- | --- |
| **ID** | **Query** |
| **#01** | MeSH descriptor: [Colorectal Surgery] explode all trees |
| **#02** | (Surgery Specialty, Colon and Rectal): ti,ab,kw OR (Colon and Rectal Surgery Specialty): ti,ab,kw OR (Colon Surgery Specialty): ti,ab,kw OR (Specialty, Colon Surgery): ti,ab,kw OR (Surgery Specialty, Colon): ti,ab,kw |
| **#03** | (Proctology): ti,ab,kw OR (Specialty, Rectal Surgery): ti,ab,kw OR (Surgery Specialty, Rectal): ti,ab,kw OR (Rectal Surgery Specialty): ti,ab,kw |
| **#04** | MeSH descriptor: [Proctectomy] explode all trees |
| **#05** | MeSH descriptor: [Colectomy] explode all trees |
| **#06** | (Proctectomies): ti,ab,kw OR (Colectomies): ti,ab,kw |
| **#07** | #1 or #2 or #3 or #4 or #5 or #6 |
| **#08** | MeSH descriptor: [Colon, Sigmoid] explode all trees |
| **#09** | MeSH descriptor: [Colon] explode all trees |
| **#10** | MeSH descriptor: [Rectum] explode all trees |
| **#11** | (Sigmoid Colon): ti,ab,kw OR (Sigmoid): ti,ab,kw OR (rectums): ti,ab,kw OR (bowel): ti,ab,kw OR (large bowel): ti,ab,kw |
| **#12** | (colonic): ti,ab,kw OR (rectal): ti,ab,kw OR (colorectal): ti,ab,kw |
| **#13** | #8 or #9 or #10 or #11 or #12 |
| **#14** | (Excision): ti,ab,kw OR (resection): ti,ab,kw OR (surgery): ti,ab,kw OR (surgical): ti,ab,kw OR (surgically): ti,ab,kw |
| **#15** | #13 and #14 |
| **#16** | #7 or #15 |
| **#17** | MeSH descriptor: [Surgical Wound Infection] explode all trees |
| **#18** | (Infections, Surgical Wound): ti,ab,kw OR (Surgical Wound Infections): ti,ab,kw OR (Wound Infections, Surgical): ti,ab,kw OR (Infection, Surgical Wound): ti,ab,kw OR (Surgical Site Infection): ti,ab,kw |
| **#19** | (Infection, Surgical Site): ti,ab,kw OR (Infections, Surgical Site): ti,ab,kw OR (Surgical Site Infections): ti,ab,kw OR (Wound Infection, Postoperative): ti,ab,kw OR (Wound Infection, Surgical): ti,ab,kw |
| **#20** | (Infection, Postoperative Wound): ti,ab,kw OR (Infections, Postoperative Wound): ti,ab,kw OR (Postoperative Wound Infections): ti,ab,kw OR (Wound Infections, Postoperative): ti,ab,kw OR (Postoperative Wound Infection): ti,ab,kw |
| **#21** | #17 or #18 or #19 or #20 |
| **#22** | MeSH descriptor: [Risk Factors] explode all trees |
| **#23** | (Factor, Risk):ti,ab,kw OR (Factors, Risk):ti,ab,kw OR (Risk Factor):ti,ab,kw OR (Population at Risk):ti,ab,kw OR (Risk, Population at):ti,ab,kw |
| **#24** | (Populations at Risk):ti,ab,kw OR (Risk, Populations at):ti,ab,kw |
| **#25** | #22 or #23 or #24 |
| **#26** | #16 and #21 and #25 |
