# Supplementary material for: Risk factors for surgical site infection in patients undergoing colorectal surgery: A meta-analysis of observational studies
Source: PLoS One. 2021 Oct 28;16(10):e0259107. doi: 10.1371/journal.pone.0259107 (PMC8553052; doi:10.1371/journal.pone.0259107)
Supplement: S2 Table — (DOC) [file pone.0259107.s002.doc]

**S2 Table.** The quality assessment of included studies by the Newcastle Ottawa scale (NOS)

| **Study** | **Selection** | | | | **Comparability** | **Outcome** | | | **Total** |
| --- | --- | --- | --- | --- | --- | --- | --- | --- | --- |
|  | Representativeness of the exposed cohort | Selection of the non-exposed cohort | Ascertainment of exposure | Demonstration that outcome of interest was not present at start of study | Comparability of cohorts on the basis of the design or analysis | Assessment of outcome | Was follow-up long enough for outcomes to occur | Adequacy of follow up of cohorts |  |
| **Kwaan2013** | ☆ | ☆ | ☆ |  | ☆ | ☆ | ☆ |  | **6** |
| **Kwaan2015** | ☆ | ☆ | ☆ |  | ☆ | ☆ | ☆ | ☆ | **7** |
| **Bot2013** | ☆ | ☆ | ☆ |  | ☆☆ | ☆ | ☆ | ☆ | **8** |
| **Bert2017** | ☆ | ☆ | ☆ |  | ☆ |  | ☆ | ☆ | **6** |
| **Poeran2016** | ☆ | ☆ | ☆ |  | ☆ | ☆ | ☆ | ☆ | **7** |
| **Guzman2018** | ☆ | ☆ | ☆ |  | ☆ | ☆ | ☆ | ☆ | **7** |
| **Ho2011** | ☆ | ☆ | ☆ |  | ☆ | ☆ | ☆ | ☆ | **7** |
| **Nakamura2008** | ☆ | ☆ | ☆ |  | ☆ | ☆ | ☆ | ☆ | **7** |
| **Hennessey2015** | ☆ | ☆ | ☆ |  | ☆ | ☆ | ☆ | ☆ | **7** |
| **Uchino2013** | ☆ | ☆ | ☆ | ☆ | ☆ | ☆ | ☆ | ☆ | **8** |
| **Tang 2001** | ☆ | ☆ | ☆ | ☆ | ☆ | ☆ | ☆ | ☆ | **8** |
| **Biondo2012** | ☆ | ☆ | ☆ |  | ☆☆ | ☆ | ☆ | ☆ | **8** |
| **Bislenghi2019** | ☆ | ☆ | ☆ | ☆ | ☆ | ☆ | ☆ | ☆ | **8** |
| **Itatsu2013** | ☆ | ☆ | ☆ | ☆ | ☆ | ☆ | ☆ | ☆ | **8** |
| **Hibbert2015** | ☆ | ☆ | ☆ | ☆ | ☆ | ☆ | ☆ | ☆ | **8** |
| **Hubner2011** | ☆ | ☆ | ☆ | ☆ | ☆ | ☆ | ☆ | ☆ | **8** |
| **Wick2011** | ☆ | ☆ | ☆ |  | ☆ | ☆ | ☆ | ☆ | **7** |
| **Blumetti2007** | ☆ | ☆ | ☆ |  | ☆ | ☆ | ☆ | ☆ | **7** |
| **Tserenpuntsag2014** | ☆ | ☆ | ☆ |  | ☆ | ☆ | ☆ | ☆ | **7** |
| **Imai2008** | ☆ | ☆ | ☆ |  | ☆ | ☆ | ☆ | ☆ | **7** |
| **Colas-Ruiz 2018** | ☆ | ☆ | ☆ | ☆ | ☆ | ☆ | ☆ | ☆ | **8** |
| **Park 2015** | ☆ | ☆ | ☆ |  | ☆ | ☆ | ☆ | ☆ | **7** |
| **Silvestri2017** | ☆ | ☆ | ☆ |  | ☆ | ☆ | ☆ | ☆ | **7** |
| **Cima2017** | ☆ | ☆ | ☆ |  | ☆ | ☆ | ☆ | ☆ | **7** |
| **Watanabe2015** | ☆ | ☆ | ☆ |  | ☆ | ☆ | ☆ | ☆ | **7** |
| **Mason2016** | ☆ | ☆ | ☆ |  | ☆ | ☆ | ☆ | ☆ | **7** |
| **Mik2016** | ☆ | ☆ | ☆ |  | ☆ | ☆ | ☆ | ☆ | **7** |
| **Olmez2019** | ☆ | ☆ | ☆ |  | ☆ | ☆ | ☆ | ☆ | **7** |
| **Uchino2009** | ☆ | ☆ | ☆ | ☆ | ☆ | ☆ | ☆ | ☆ | **8** |
| **Ghuman2015** | ☆ | ☆ | ☆ |  | ☆ | ☆ | ☆ | ☆ | **7** |
| **Poon 2009** | ☆ | ☆ | ☆ | ☆ | ☆☆ | ☆ | ☆ | ☆ | **9** |
