# Supplementary material for: Risk factors for surgical site infection in patients undergoing colorectal surgery: A meta-analysis of observational studies
Source: PLoS One. 2021 Oct 28;16(10):e0259107. doi: 10.1371/journal.pone.0259107 (PMC8553052; doi:10.1371/journal.pone.0259107)
Supplement: S3 Table — (DOC) [file pone.0259107.s003.doc]

| **S3 Table.** The relevant risk factors of incision surgical site infection | | |
| --- | --- | --- |
| **Study** | **Risk factors** |  |
| **Kwaan 2013** | Prophylactic antibiotic not administered in a timely manner (OR=4.33, 95%CI: 1.08-17.4) | |
| **Uchino 2013** | Ostomy creation (OR=3.45, 95%CI: 1.80-6.62); Operative time ≥167 min (OR=2.17, 95%CI: 1.16-4.05); Proctectomy (OR=5.37, 95%CI: 2.73-10.54) | |
| **Tang 2001** | ASA score 2 (Ref. ASA score 1) (OR=1.9, 95%CI: 1.1-3.2); Albumin＜3 g (OR=2.3, 95%CI: 1.1-4.7); Ostomy creation (OR=2.9,95%CI: 1.6-5.3); Operative time＞180min (OR=2.6, 95%CI: 1.4-4.8); Wound class 3(Ref. wound class 2) (OR=4.0, 95%CI: 1.6-8.9) | |
| **Biondo 2012** | Blood transfusion (OR=2.036, 95%CI: 1.459–2.842); Tumor Stage III (OR=1.572, 95%CI: 1.037–2.385); Tumor Stage IV (OR=1.797, 95%CI: 1.121–2.879) | |
| **Itatsu 2013** | Previous laparotomy (OR=1.56, 95%CI: 1.11-2.19); Chronic liver disease (OR=2.43, 95%CI: 1.17-5.05); Wound length ≥20cm (Ref.＜20cm) (OR=1.68, 95%CI: 1.20-2.36); Wound class 3 (Ref. wound class≤2) (OR=3.23, 95%CI: 1.52-6.83); Ostomy creation (OR=2.29, 95%CI: 1.17-4.50) | |
| **Poon 2009** | Open surgery (OR=2.36, 95%CI: 1.05-5.3); Blood transfusion (OR=2.43, 95%CI: 1.0-5.9); Operative time (OR=1.13, 95%CI: 0.9-1.38); Anastomotic leakage (OR=6.5, 95%CI: 2.3-18.6) | |
| **Liu 2018** | Anemia (OR=2.44, 95%CI: 1.09-5.49); Ostomy creation (OR=2.64, 95%CI: 1.2-5.81) | |
| **Ishikawa 2014** | TNM stages III and IV (Ref. I and II ) (OR=2.4, 95%CI: 1.1-5.80); Intraoperative hypotension SBP < 80 mmHg (OR=3.4, 95%CI: 1.3-10.7) | |
| **Sergeant 2008** | ASA-score≤2 (OR=0.60, 95%CI: 0.21-1.41); COPD (OR=2.53, 95%CI: 1.19-5.83); Operation time (OR=1.02, 95%CI: 1.01 - 1.04) | |
| **Ge 2019** | Laparoscopic surgery (OR=0.374, 95%CI: 0.152-0.92); Ostomy creation (OR=1.419, 95%CI: 0.631-3.193);Wound protector (OR=0.357, 95%CI: 0.161-0.793); Repeated surgery (OR=0.411, 95%CI: 0.193-0.876) | |
| **Cima 2017** | S-SSI: BMI ≥30 (Ref. <30/unknown) (OR=2.14, 95%CI: 1.40–3.29); ASA III-IV (Ref. I-II) (OR=1.69, 95%CI: 1.08–2.64); Steroid use (Ref. no) (OR=1.72, 95%CI: 1.01–2.93) | |
| **Nasser 2020** | S-SSI: Smoking (OR: 1.29, 95%CI: 1.16-1.44); BMI for every 5 kg/m2 increase (OR=1.24, 95%CI: 1.20-1.27); ASA class≥3 (OR=1.21, 95%CI: 1.11-1.32); Wound class 3 (Ref. wound class≤2) (OR=1.21, 95%CI: 1.07-1.37); Steroid use (OR=1.19, 95%CI: 1.03-1.37); Operative time for every 60 min increase (OR=1.12, 95%CI: 1.09-1.15) | |
|  | D-SSI: Steroid use (OR=1.81, 95%CI: 1.31-2.49) Smoking (OR=1.50, 95%CI: 1.17-1.94); ASA class≥3 (OR=1.41, 95%CI: 1.13-1.76) | |
| **Mik 2016** | S-SSI: Age>65 (Ref. ≤65) (OR=0.68, 95%CI: 0.62–0.74);Albumin<35 mg/ml (OR=1.61, 95%CI:1.58–1.64);Respiratory disease (OR=1.29, 95%CI:1.24–1.33); Emergent surgery (OR=1.46, 95%CI: 1.43–1.50) | |
|  | D-SSI: Age>65 (Ref. ≤65) (OR=0.84, 95%CI: 0.80–0.90); Albumin<35 mg/ml (OR=1.12, 95%CI: 1.10–1.15); Protective stoma (NO versus Yes) (OR=1.16, 95%CI: 1.13–1.20); Emergent surgery (OR=1.29, 95%CI: 1.23–1.28) | |
| **Segal 2014** | S-SSI: Age in 5 y increments (OR=0.98, 95%CI: 0.97–0.99); Operative time (OR=1.14, 95%CI: 1.11–1.16); Laparoscopic surgery (OR=0.66, 95%CI: 0.61–0.71); Wound class >2 (OR=1.17, 95%CI: 1.08–1.27); ASA class >2 (OR=1.18, 95%CI: 1.09–1.27); Diverticulitis(vs neoplasms) (OR=1.13, 95%CI: 1.03–1.24); Bowel obstruction/perforation (OR=1.21, 95%CI: 1.06–1.39); Diabetes mellitus (OR=1.11, 95%CI: 1.02–1.21); Alcohol >2 drinks/d (OR=1.22, 95%CI: 1.04–1.42); COPD (OR=1.14, 95%CI: 1.00–1.29); Preoperative dyspnea (OR=1.13, 95%CI: 1.03–1.25) | |
|  | D-SSI: Operative time (OR=1.24, 95%CI:1.20–1.29); Laparoscopic surgery (OR=0.60, 95%CI: 0.50–0.72); Wound class >2 (OR=1.31, 95%CI: 1.12–1.54); ASA class >2 (OR=1.46, 95%CI: 1.24–1.71); Enteritis/colitis(vs neoplasms) (OR=1.45, 95%CI: 1.05–1.20); Bowel obstruction/perforation (OR=1.75, 95%CI: 1.35–2.27); Smoker within 1 year of surgery (OR=1.28, 95%CI: 1.09–1.52) | |
| **Kohut 2015** | S-SSI: Operative time＞180 min (OR=1.41, 95%CI: 1.31-1.52); ASA class≥3 (OR=1.25, 95%CI:1.16-1.35); COPD (OR=1.26, 95%CI: 1.10-1.45); Wound class >2 (OR=1.29, 95%CI: 1.18–1.40); Smoking (OR=1.15, 95%CI: 1.06-1.26) | |
|  | D-SSI: Operative time＞180min (OR=1.74, 95%CI: 1.47-2.05); ASA class≥3 (OR=1.40, 95%CI: 1.18-1.66); Wound class >2 (OR=1.22, 95%CI: 1.01–1.49); History of CHF (OR=1.74, 95%CI: 1.00-3.04); COPD (OR=1.41, 95%CI: 1.05-1.87); Smoking (OR=1.27, 95%CI: 1.05-1.54) | |
