# Supplementary material for: Risk factors for surgical site infection in patients undergoing colorectal surgery: A meta-analysis of observational studies
Source: PLoS One. 2021 Oct 28;16(10):e0259107. doi: 10.1371/journal.pone.0259107 (PMC8553052; doi:10.1371/journal.pone.0259107)
Supplement: S4 Table — (DOC) [file pone.0259107.s004.doc]

**S4 Table.** Sensitivity analysis of the meta-analysis

| **Risk factors** | **Fixed-effects model** | **Random-effects model** |
| --- | --- | --- |
| **Obesity** | 1.60 [1.54, 1.66] | 1.61 [1.51, 1.70] |
| **Male sex** | 1.25 [1.16, 1.35] | 1.30 [1.14, 1.49] |
| **Diabetes mellitus** | 1.16 [1.10, 1.23] | 1.65 [1.24, 2.20] |
| **Lung disease** | 1.23 [1.15, 1.30] | 2.62 [0.84, 8.13] |
| **ASA score≧3** | 1.34 [1.19, 1.51] | 1.34 [1.19, 1.51] |
| **Cigarette smoking** | 1.24 [1.18, 1.30] | 1.38 [1.14, 1.67] |
| **Wound classification** | 1.22 [1.15, 1.30] | 2.65 [1.52, 4.61] |
| **Neoplasm** | 0.70 [0.64, 0.77] | 1.24 [0.58, 2.66] |
| **Inflammatory bowel disease** | 1.99 [1.45, 2.73] | 2.12 [1.24, 3.61] |
| **Open surgery** | 1.79 [1.72, 1.87] | 1.81 [1.57, 2.10] |
| **Stoma creation** | 1.24 [1.18, 1.30] | 1.38 [1.14, 1.67] |
| **Emergent surgery** | 1.34 [1.30, 1.38] | 1.36 [1.19, 1.55] |
| **Blood transfusion** | 1.80 [1.53, 2.12] | 2.03 [1.34, 3.06] |
| **Operative time (≧180min)** | 1.64 [1.59, 1.70] | 1.88 [1.49, 2.36] |
