# Supplementary material for: Risk factors for surgical site infection in patients undergoing colorectal surgery: A meta-analysis of observational studies
Source: PLoS One. 2021 Oct 28;16(10):e0259107. doi: 10.1371/journal.pone.0259107 (PMC8553052; doi:10.1371/journal.pone.0259107)
Supplement: S5 Table — (DOC) [file pone.0259107.s005.doc]

**S5 Table.** Sensitivity analysis of the meta-analysis

| **Risk factor** | **Study omitted** | **OR** | **OR_LL** | **OR_UL** | **I^2^** | **P value (Cochrane Q)** |
| --- | --- | --- | --- | --- | --- | --- |
| **Obesity** | None | 1.60 | 1.47 | 1.74 | 25% | 0.23 |
|  | Bislenghi2019 | 1.60 | 1.46 | 1.75 | 33% | 0.18 |
|  | Bot2013 | 1.60 | 1.46 | 1.75 | 32% | 0.18 |
|  | Cima2017 | 1.60 | 1.45 | 1.77 | 34% | 0.17 |
|  | Hibbert2015 | 1.58 | 1.51 | 1.65 | 0% | 0.97 |
|  | Mik2016 | 1.65 | 1.43 | 1.90 | 35% | 0.16 |
|  | Poeran2016 | 1.62 | 1.45 | 1.81 | 35% | 0.16 |
|  | Tserenpuntsag2014 | 1.63 | 1.47 | 1.82 | 32% | 0.19 |
|  | Wick 2011 | 1.62 | 1.45 | 1.81 | 35% | 0.16 |
| **Male sex** | None | 1.30 | 1.14 | 1.49 | 59% | 0.02 |
|  | Biondo2012 | 1.30 | 1.11 | 1.51 | 64% | 0.01 |
|  | Hubner2011 | 1.27 | 1.10 | 1.46 | 57% | 0.03 |
|  | Imai2008 | 1.31 | 1.13 | 1.51 | 65% | 0.009 |
|  | Mason2016 | 1.28 | 1.13 | 1.46 | 57% | 0.03 |
|  | Poeran2016 | 1.33 | 1.11 | 1.60 | 65% | 0.009 |
|  | Tang2001 | 1.29 | 1.11 | 1.49 | 63% | 0.01 |
|  | Tserenpuntsag2014 | 1.30 | 1.11 | 1.53 | 64% | 0.01 |
|  | Wick2011 | 1.34 | 1.23 | 1.47 | 0% | 0.50 |
| **Diabetes mellitus** | None | 1.65 | 1.24 | 2.20 | 60% | 0.01 |
|  | Bot2013 | 1.71 | 1.23 | 2.38 | 64% | 0.006 |
|  | Cima2017 | 1.54 | 1.16 | 2.04 | 47% | 0.07 |
|  | Ghuman2015 | 1.55 | 1.18 | 2.03 | 53% | 0.04 |
|  | Guzman-Pruneda2018 | 1.62 | 1.20 | 2.19 | 62% | 0.01 |
|  | Imai2008 | 1.71 | 1.25 | 2.36 | 65% | 0.006 |
|  | Kwaan2015 | 1.86 | 1.47 | 2.35 | 0% | 0.78 |
|  | Mason2016 | 1.66 | 1.23 | 2.24 | 65% | 0.006 |
|  | Park2015 | 1.60 | 1.19 | 2.15 | 59% | 0.02 |
|  | Silvestri2017 | 1.63 | 1.19 | 2.22 | 60% | 0.01 |
| **Lung disease** | None | 2.62 | 0.84 | 8.13 | 76% | 0.02 |
|  | Bislenghi2019 | 3.02 | 0.35 | 26.00 | 81% | 0.02 |
|  | Kwaan2015 | 4.66 | 1.46 | 14.89 | 25% | 0.25 |
|  | Park2015 | 1.68 | 0.70 | 4.03 | 67% | 0.08 |
| **Cigarette smoking** | None | 1.38 | 1.14 | 1.67 | 64% | 0.02 |
|  | Cima2017 | 1.38 | 1.11 | 1.73 | 69% | 0.01 |
|  | Ghuman2015 | 1.27 | 1.11 | 1.45 | 42% | 0.14 |
|  | Guzman-Pruneda2018 | 1.28 | 1.09 | 1.51 | 55% | 0.06 |
|  | Kwaan2015 | 1.75 | 1.14 | 2.68 | 71% | 0.008 |
|  | Mason2016 | 1.38 | 1.13 | 1.69 | 71% | 0.009 |
|  | Poeran2016 | 1.74 | 1.19 | 2.57 | 68% | 0.01 |
| **Wound classification** | None | 2.65 | 1.52 | 4.61 | 86% | ﹤0.00001 |
|  | Bert2017 | 2.93 | 1.36 | 6.29 | 86% | ﹤0.00001 |
|  | Ho2011 | 2.29 | 1.34 | 3.93 | 85% | ﹤0.00001 |
|  | Itatsu2013 | 2.56 | 1.41 | 4.65 | 86% | ﹤0.00001 |
|  | Kwaan2013 | 2.64 | 1.46 | 4.76 | 88% | ﹤0.00001 |
|  | Kwaan2015 | 3.15 | 1.97 | 5.04 | 49% | 0.08 |
|  | Silvestri2017 | 2.81 | 1.53 | 5.16 | 88% | ﹤0.00001 |
|  | Watanabe2015 | 2.24 | 1.33 | 3.78 | 80% | 0.0001 |
| **Neoplasm** | None | 1.24 | 0.58 | 2.66 | 81% | 0.0003 |
|  | Bislenghi2019 | 1.67 | 0.84 | 3.31 | 41% | 0.17 |
|  | Colas-Ruiz2018 | 1.19 | 0.53 | 2.69 | 85% | 0.0002 |
|  | Guzman-Pruneda2018 | 1.05 | 0.45 | 2.41 | 77% | 0.004 |
|  | Mason2016 | 1.51 | 0.60 | 3.78 | 85% | 0.0001 |
|  | Poeran2016 | 0.98 | 0.47 | 2.05 | 66% | 0.03 |
| **Inflammatory bowel disease** | None | 2.12 | 1.24 | 3.61 | 63% | 0.07 |
|  | Cima2017 | 2.51 | 1.02 | 6.19 | 76% | 0.04 |
|  | Poeran2016 | 1.63 | 1.14 | 2.34 | 0% | 0.98 |
|  | Uchino2009 | 2.50 | 1.03 | 6.09 | 78% | 0.03 |
| **Open surgery** | None | 1.81 | 1.57 | 2.10 | 69% | ﹤0.0001 |
|  | Bert2017 | 1.83 | 1.58 | 2.13 | 71% | ﹤0.0001 |
|  | Biondo2012 | 1.89 | 1.65 | 2.18 | 63% | 0.0006 |
|  | Bot2013 | 1.71 | 1.50 | 1.94 | 58% | 0.003 |
|  | Cima2017 | 1.85 | 1.59 | 2.16 | 70% | ﹤0.0001 |
|  | Guzman-Pruneda2018 | 1.77 | 1.54 | 2.03 | 66% | 0.0001 |
|  | Hennessey2015 | 1.85 | 1.60 | 2.14 | 70% | ﹤0.0001 |
|  | Ho2011 | 1.84 | 1.59 | 2.14 | 70% | ﹤0.0001 |
|  | Hubner2011 | 1.76 | 1.51 | 2.04 | 68% | ﹤0.0001 |
|  | Imai2008 | 1.83 | 1.57 | 2.13 | 71% | ﹤0.0001 |
|  | Kwaan2015 | 1.88 | 1.54 | 2.30 | 70% | ﹤0.0001 |
|  | Nakamura2008 | 1.79 | 1.55 | 2.08 | 70% | ﹤0.0001 |
|  | Olmez2019 | 1.79 | 1.55 | 2.07 | 70% | ﹤0.0001 |
|  | Park2015 | 1.88 | 1.56 | 2.26 | 70% | ﹤0.0001 |
|  | Poon2009 | 1.88 | 1.55 | 2.09 | 71% | ﹤0.0001 |
|  | Watanabe2015 | 1.81 | 1.56 | 2.10 | 71% | ﹤0.0001 |
|  | Wick2011 | 1.86 | 1.58 | 2.18 | 70% | ﹤0.0001 |
| **Stoma creation** | None | 1.89 | 1.28 | 2.78 | 69% | 0.002 |
|  | Blumetti2007 | 1.87 | 1.17 | 2.99 | 73% | 0.001 |
|  | Bot2013 | 1.97 | 1.30 | 3.01 | 72% | 0.002 |
|  | Guzman-Pruneda2018 | 2.01 | 1.33 | 3.01 | 71% | 0.002 |
|  | Ho2011 | 2.21 | 1.61 | 3.03 | 46% | 0.09 |
|  | Itatsu2013 | 1.83 | 1.18 | 2.84 | 73% | 0.001 |
|  | Tang2001 | 1.84 | 1.16 | 2.92 | 73% | 0.001 |
|  | Uchino2009 | 1.72 | 1.14 | 2.59 | 67% | 0.006 |
|  | Uchino2013 | 1.70 | 1.16 | 2.49 | 63% | 0.01 |
| **Emergent surgery** | None | 1.36 | 1.19 | 1.55 | 40% | 0.12 |
|  | Bert2017 | 1.39 | 1.22 | 1.58 | 37% | 0.16 |
|  | Blumetti2007 | 1.33 | 1.18 | 1.49 | 32% | 0.19 |
|  | Hennessey2015 | 1.36 | 1.18 | 1.58 | 50% | 0.07 |
|  | Hubner2011 | 1.33 | 1.14 | 1.56 | 45% | 0.10 |
|  | Mik2016 | 1.43 | 1.12 | 1.83 | 50% | 0.07 |
|  | Poeran2016 | 1.44 | 1.18 | 1.76 | 43% | 0.12 |
|  | Silvestri2017 | 1.33 | 1.19 | 1.48 | 29% | 0.23 |
| **Blood transfusion** | None | 2.03 | 1.34 | 3.06 | 74% | 0.004 |
|  | Biondo2012 | 1.87 | 1.14 | 3.06 | 54% | 0.09 |
|  | Colas-Ruiz2018 | 2.09 | 1.33 | 3.28 | 80% | 0.002 |
|  | Park2015 | 1.85 | 1.18 | 2.91 | 76% | 0.005 |
|  | Poon2009 | 1.98 | 1.24 | 3.15 | 80% | 0.002 |
|  | Tserenpuntsag2014 | 2.47 | 1.96 | 3.11 | 0% | 0.81 |
| **Operative time** | None | 1.88 | 1.49 | 2.36 | 58% | 0.04 |
|  | Bislenghi2019 | 1.82 | 1.44 | 2.29 | 61% | 0.04 |
|  | Hennessey2015 | 1.64 | 1.59 | 1.69 | 0% | 0.58 |
|  | Hubner2011 | 2.24 | 1.49 | 3.39 | 65% | 0.02 |
|  | Mik2016 | 2.22 | 1.45 | 3.41 | 64% | 0.03 |
|  | Olmez2019 | 1.94 | 1.50 | 2.50 | 66% | 0.02 |
|  | Park2015 | 1.86 | 1.45 | 2.38 | 64% | 0.03 |
