# Supplementary material for: Risk factors for surgical site infection in patients undergoing colorectal surgery: A meta-analysis of observational studies
Source: PLoS One. 2021 Oct 28;16(10):e0259107. doi: 10.1371/journal.pone.0259107 (PMC8553052; doi:10.1371/journal.pone.0259107)
Supplement: S1 Fig — (DOC) [file pone.0259107.s006.doc]

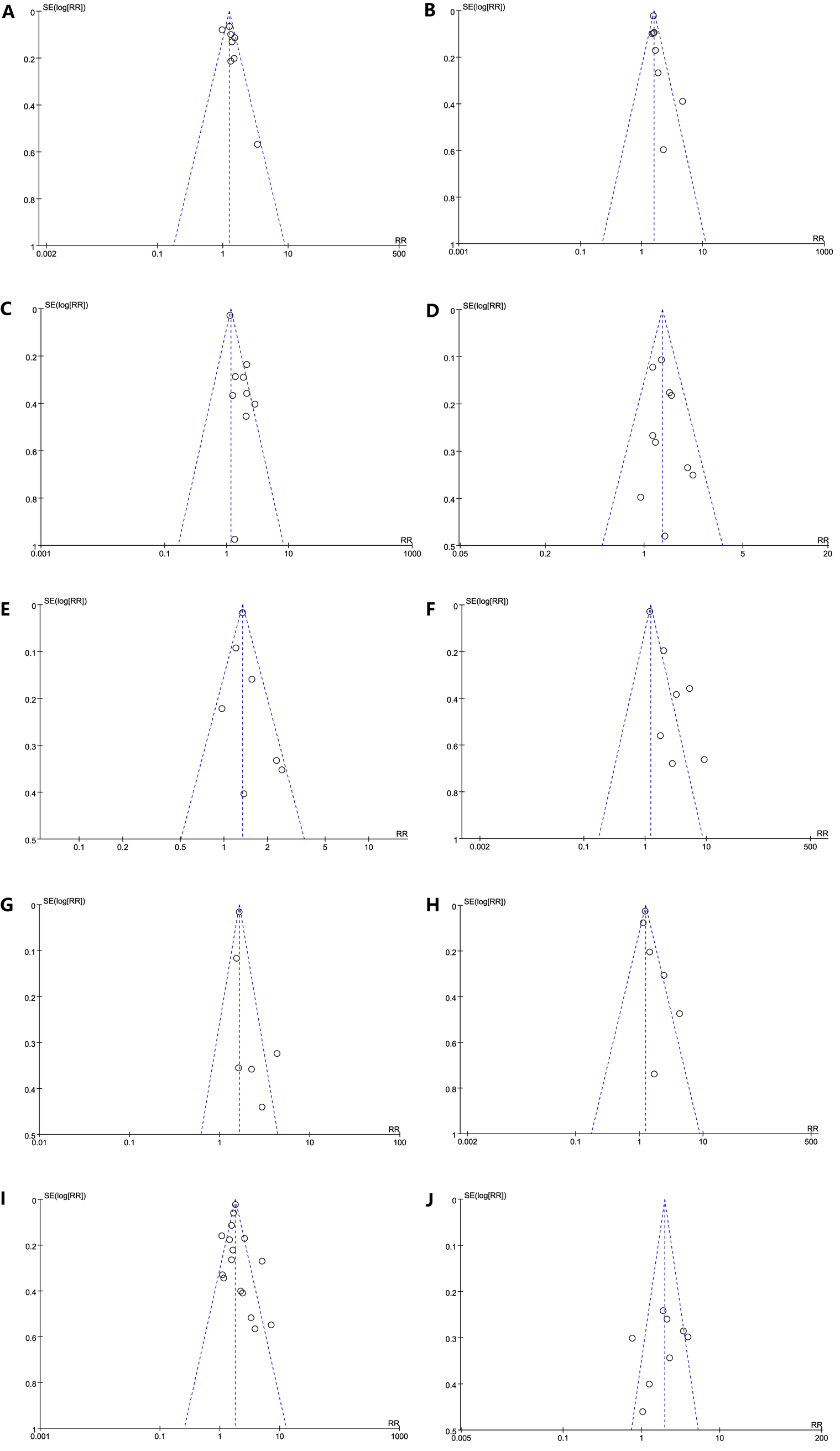


**S1 Fig**. The publication bias of the relevant factors of SSIs (A: male sex; B: obesity; C: diabetes mellitus; D: ASA score≥3; E: emergent surgery; F: wound classification> 2; G: operative time ≥180 min; H: cigarette smoking; I: open surgery; J: stoma formation).
