# Supplementary figures and images for: Risk factors for surgical site infection in patients undergoing colorectal surgery: A meta-analysis of observational studies
Source: PLoS One. 2021 Oct 28;16(10):e0259107. doi: 10.1371/journal.pone.0259107 (PMC8553052; doi:10.1371/journal.pone.0259107)

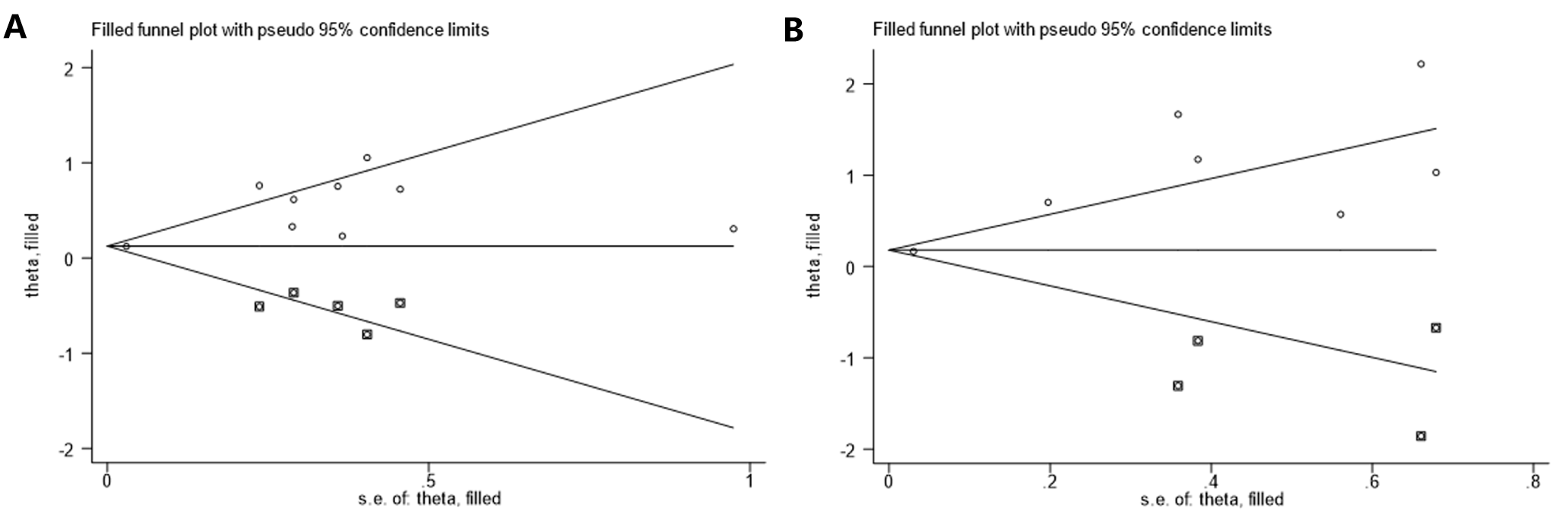
**S2 Fig**. The trim and fill graphs. (A: Diabetes mellitus; B: wound classification>2)

Supplement: S2 Fig — (A: Diabetes mellitus; B: wound classification>2). (DOC) [file pone.0259107.s007.doc]
